# Comparison of interleukin-21 levels and its correlation with clinical parameters among healthy individuals, chronic periodontitis, and aggressive periodontitis patients

**Published:** 2021-02-02

**Authors:** Archana Mootha, Sankari Malaiappan, Dan M. J. Milstein, Gurumoorthy Karthikeyan, Sheeja S. Varghese, N. Doraisamy Jayakumar

**Affiliations:** ^1^Graduate school of Advanced Sciences of Matter, Hiroshima University, Hiroshima, Japan; ^2^Department of Periodontics, Saveetha Dental College and Hospitals, Chennai, Tamil Nadu, India; ^3^Department of Oral Medicine, Academic Centre for Dentistry Amsterdam, University of Amsterdam and VU University Amsterdam, Amsterdam, The Netherlands; ^4^Department of Periodontics, Saveetha Dental College and hospitals, Chennai, Tamil Nadu, India

**Keywords:** Adult periodontitis, aggressive periodontitis, cytokine, IL-21, inflammation

## Abstract

**Background:**

Interleukin-21 (IL-21) has both anti- and pro-inflammatory roles associated with chronic inflammation. It causes tissue destruction by increasing pro-inflammatory cytokines and curbs the activity of certain immune cells that evoke an anti-inflammatory role.

**Objectives:**

The aim of this study was to compare IL-21 levels in gingival crevicular fluid among patients with generalized chronic periodontitis (GCP), aggressive periodontitis, and healthy gingiva (HG) and to correlate IL-21 levels with clinical parameters.

**Methods:**

In this cross-sectional case-control study, 60 subjects were categorized into three groups: HG (*n*=20), generalized aggressive periodontitis (GAP; *n*=20), and GCP (*n*=20). IL-21 was measured using ELISA and results were correlated with clinical parameters including plaque index, gingival index, periodontal probing depth (PPD), and clinical attachment level (CAL).

**Results:**

Mean IL-21 levels were 20.0±0.7 in HG, 25.9±0.9 in GCP, and 25.3±1.1 in GAP groups. Significant differences in IL-21 levels were found between HG versus GAP (*P*<0.05) and HG versus GCP (*P*<0.05). No statistically significant difference in IL-21 level was found between GCP versus GAP. IL-21 levels positively correlated with PPD (*r*=0.97) and CAL (*r*=0.93) in the GAP group and with PPD (*r*=0.92) and CAL (*r*=0.96) in the GCP group.

**Conclusion:**

Although periodontitis pathophysiology involves complex interplay between pro- and anti-inflammatory signaling, data on IL-21 revealed elevated levels in both GCP and GAP. Further longitudinal studies are required to characterize and determine the diagnostic value of IL-21 as a reliable biomarker in periodontal disease.

**Relevance for patients:**

Although further longitudinal studies are necessary, IL-21 may serve as a potential inflammatory biomarker in screening for generalized chronic and aggressive periodontitis.

## 1. Introduction

Periodontitis is an inflammatory disease of the supporting structures of teeth. It is usually caused by a dysbiotic subgingival microbial community from dental plaque along with genetic factors, resulting in progressive periodontal breakdown that manifests clinically as pocket formation and alveolar bone loss [1,2]. Periodontitis is one of the most common reasons for tooth loss and 10-15% of the general adult population is affected by severe forms of periodontitis [3,4]. The host responds to this dysbiosis with an immunoinflammatory reaction mediated by secretion of inflammatory mediators such as cytokines, prostaglandins, and matrix metalloproteinases (MMPs) [5-8]. These inflammatory mediators provide the signals to innate immune cells to act against the virulent factors of invading pathogens and the non-specific innate immune mechanism counteracts microbial invasion to an extent. Chronic inflammation leads to activation of the adaptive immune system. T cells play a central role in the adaptive immune response and among the various T cells, T helper (Th) cells are arguably the most distinctive cell types [9]. Once there is a halt in further bone and soft-tissue destruction, the immune response is maintained by a balance between Th1- and Th2-mediated roles. A novel subset of Th cells, Th17 was identified which mainly produces interleukin (IL)-17, IL-21, IL-22, and IL-23 [10]. It was observed that progressive periodontal lesions are characterized by Th cells that belong to Th1 and Th17 subsets and stable periodontal lesions contain Th2 cells and their respective cytokine profiles [11].

**Figure 1:**
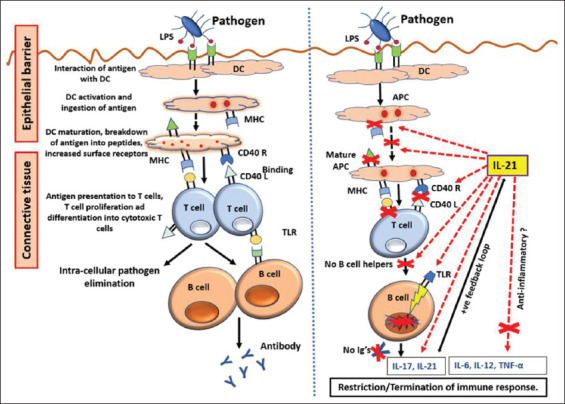
Molecular and cellular immunopathogenesis involving IL-21. IL: Interleukin, DC: Dendritic cell, MHC: Major histocompatibility complex, TLR: T-cell receptor, APC: Antigen-presenting cell, Ig: Immunoglobulin, TGF-α: Transforming growth factor-alpha.

IL-21 is an inflammatory cytokine involved in innate and adaptive immune responses and is mainly expressed by distinct activated pro-inflammatory lineages associated with Th1 and Th17 [12]. IL-21 has a dual role where it alleviates and aggravates specific immune responses in host defense. It aggravates the immune response by activating other immune cells to further produce inflammatory cytokines [13]. IL-21 activates natural killer cells and promotes the migration of polymorphonuclear neutrophils (PMNs) to the site of inflammation and also aids in the terminal conversion of B cells into plasma cells. IL-21 also induces either Th1/Th2 cell differentiation based on the immunological context and alleviates inflammatory responses by inhibiting B cell proliferation and dendritic cell activation in the presence of bacterial lipopolysaccharide [14]. Experimental studies have reported that IL-21 favors apoptosis of Toll-like receptor activated B cells [15] and that it balances out local cytokine expressions by increasing IL-10, decreasing TNF-α production, and regulates the generation of pro-inflammatory cytokines such as IL-17 and IL-6 by regulatory T cells (Treg) [11,16,17] (Figure 1).

The endogenous production of IL-21 is biologically significant because the number of IL-17 producing cells significantly decreases in the absence of IL-21 due to the dearth of positive feedback loop. Along these lines, IL-21 can be speculated to make a distinct contribution to the progression of inflammation [17]. Much controversy exists regarding classification of periodontitis cases into chronic or aggressive types. The specific clinical characteristics and the association of specific microbial complexes or serotypes of specific pathogens with aggressive or chronic periodontitis have been established by many studies [18-20]. Similarly, a distinct difference in host immune inflammatory response to the bacterial dysbiosis could contribute to the difference between chronic and aggressive periodontitis [21,22]. Differences in host immune inflammatory responses associated with chronic and aggressive periodontitis include genetic factors, functional defects of immune cells such as PMNs, and differences in inflammatory mediators.

Gingival crevicular fluid (GCF) is an inflammatory exudate that flows into and out of the periodontal pocket and can be used to evaluate the state of local tissue destruction and the relationship between systemic diseases and the periodontium. Although data derived from GCF levels of various inflammatory mediators like cytokines/chemokines in aggressive periodontitis subjects and chronic periodontitis subjects differ, many studies have found a significant increase in levels of pro-inflammatory cytokine/chemokines such as IL-6, IL-8, IL-1β, IL-17, TNF-α, and IL-29 in aggressive periodontitis compared to chronic periodontitis cases [23-27]. The most important finding was that anti-inflammatory cytokines IL-2, IL-4, and IL-11 levels in GCF of aggressive periodontitis cases were lower compared to chronic periodontitis subjects [28,29]. Thus, there is some evidence that supports the increased level of pro-inflammatory cytokines and reduced level of anti-inflammatory cytokines in GCF of aggressive periodontitis subjects compared to chronic periodontitis subjects [30]. A recent study by Gumus *et al*. showed an increased level of IL-21 in saliva of aggressive periodontitis patients compared to chronic periodontitis [31].

Recently, a systematic review reported elevated IL-21 levels in chronic periodontitis patients compared to healthy controls [32]. The systematic review included cross-sectional studies quantifying IL-21 in GCF, saliva, and gingival tissues along with a study reporting reduction in IL-21 levels in chronic periodontitis following non-surgical periodontal therapy [33-36]. It is well-known that a GCF sample is more specific and sensitive assay for periodontal destruction compared to saliva. However, there are no studies in the literature quantifying GCF IL-21 levels from aggressive periodontitis or studies comparing the GCF levels of IL-21 in aggressive and chronic periodontitis [37]. Thus, the aim of this study was to compare IL-21 levels in GCF among patients with generalized chronic periodontitis (GCP), aggressive periodontitis, and healthy gingiva (HG) and to correlate IL-21 levels with clinical parameters.

## 2. Materials and Methods

This cross-sectional case-control study was conducted over a period of 6 months (December 2015-May 2016) at the Department of Periodontics of the Saveetha Dental College and Hospitals of Saveetha University in Chennai, India. The study protocol was reviewed and approved by the university scientific review board and ethical committee (IHEC/SDMDS13PER2). Each participant received detailed information regarding the study procedure and a written informed consent was obtained from those who agreed to participate voluntarily in this study.

### 2.1. Study participants and clinical parameters

Patients clinically diagnosed with generalized chronic and generalized aggressive periodontitis (GAP) attending the outpatient section of the Department of Periodontics of the Saveetha Dental College and Hospitals in Chennai, India, were invited for participation. Each subject underwent full mouth periodontal probing and charting, along with a radiological investigation. Periodontal parameters including plaque index (PI), gingival index (GI), probing depth (PD), and clinical attachment level (CAL) were recorded using a UNC-15 probe (Hu-Friedy Manufacturing Co., Chicago, IL, USA). PD was measured from the gingival margin to the base of the crevice/pocket at six sites of all teeth except third molars. Periodontal attachment level was measured from the cementoenamel junction (CEJ) to the base of the crevice/pocket at the respective sites. The average of the values of these six sites was taken as a recording for that tooth. Sites with both PD of >6 mm and attachment level of >5 mm were defined as a severe site. Radiographic bone loss was also recorded.

The study included a control group with HG, GAP group, and GCP group. The diagnostic criteria for GAP and GCP were defined according to the International Workshop for a Classification of Periodontal Disease and Conditions [38]. The GAP group included subjects who had a minimum of three teeth other than first molars and incisors with PD and CAL of >6 mm and the amount of bone destruction was inconsistent with the amount of plaque and calculus on the teeth. In the GCP group, subjects had a minimum of 30% of sites with PD and CAL of >6 mm and the amount of bone destruction consistent with the amount of plaque and calculus on the teeth. The criteria for HG in control subjects included the distance between CEJ and alveolar bone not exceeding 1.5 mm on radiographs, PD of ≤3 mm, and no bleeding on probing. All subjects had at least ≥14 teeth present in each arch during the study period. Subjects with systemic diseases with periodontal manifestations, obesity, any systemic infections, immunocompromised patients, pregnant, lactating mothers, smokers, and subjects who received periodontal therapy in the past year, steroids, antibiotics, or nonsteroidal anti-inflammatory drugs within 6 months were excluded from this study.

### 2.2. GCF sample collection

The clinical examination, site selection for procurement of sample, and sample collection were performed by a single examiner. After accommodating the subjects comfortably in an upright position in a dental chair, the tooth with the maximum PD and periodontal destruction was selected. Supragingival plaque was first removed without disturbing the gingival margin and the site was isolated using cotton rolls. GCF was collected using 1-5 mL calibrated volumetric microcapillary tubes by placing it at the entrance of the gingival sulcus and gently touching the gingival margin for 5-20 min, awaiting passive flow into the tubes. A standardized volume of 1 mL GCF was collected from the test site in the periodontitis groups. The test sites that did not express 1 mL of GCF within 5-20 min and sites with blood/salivary/plaque contamination were excluded from the study. In healthy controls, GCF was collected from multiple sites and pooled. Following collection, the samples were diluted with 199 mL of phosphate buffered saline in Eppendorf tubes and stored at –70°C until further analysis.

### 2.3. Determination of IL-21 in GCF samples by ELISA

GCF samples were analyzed for IL-21 using a commercially available ELISA kit (Legend Max, BioLegend, San Diego, CA, USA). Analysis was performed according to the manufacturer’s instructions. All ELISA determinations were performed in duplicate for each sample. Briefly, 100 μL of human IL-21 detection antibody solution was added to each well and incubated at room temperature for 1 h while shaking. After the plates were washed, 100 μL of sample solution was then added to each well and incubated in the dark for 30 min. Reactions were stopped by adding 100 μL of stop solution to each well. Color change was observed from blue to yellow. Absorbance was then read immediately at 450 nm using an absorbance spectrum reader (Synergy H4 Hybrid Reader, BioTek Instruments, Winooski, VT, USA) within 30 min. Results were calculated using the standard curves included with the assay kit. The total amount of IL-21 was determined in picograms and calculations of the concentration in each sample were performed by dividing the total amount of IL-21 by the volume of the sample.

### 2.4. Statistical analysis

Using a one-way analysis of variance (ANOVA) data analysis approach for comparing three groups, with a power of 95%, an estimated effect size of 0.53, and an alpha of 0.05, a total sample size of 60 (20/group) was computed. IL-21 levels were compared between groups using one-way ANOVA and a Bonferroni *post hoc* test was used for multiple pairwise comparisons between the groups. Pearson’s correlation was used to evaluate correlations between IL-21 and clinical parameters. *P*<0.05 was considered statistically significant. Data analysis was performed using SPSS Statistics software (IBM SPSS Statistics version 25, IBM Corporation, Armonk, NY, USA).

## 3. Results

### 3.1. Clinical analysis

A total of 60 participants (28 males and 32 females) aged between 27 and 65 years were included. Demographic data and data of clinical parameters of subjects included in the study are grouped in Table 1.

**Table 1:** Demographic characteristics and clinical parameters presented as mean±SD

	HG (*n*=20)	GAP (*n*=20)	GCP (*n*=20)
Age (years)	36±5	32±6	39±9
Male: female	08:12	10:10	10:10
PI	0.2±0.4	0.5±0.5	2.8±0.4
GI	0.1±0.4	0.7±0.6	2.9±0.4
PD (mm)	1.9±0.6	9.3±1.4	9.6±0.6
CAL (mm)	0.0±0.0	9.5±1.6	9.6±0.6

HG: Healthy gingiva, GAP: Generalized aggressive periodontitis, GCP: Generalized chronic periodontitis, SD: Standard deviation, PI: Plaque index, GI: Gingival index, PD: Probing depth, CAL: Clinical attachment level.

### 3.2. GCF IL-21

**Figure 2:**
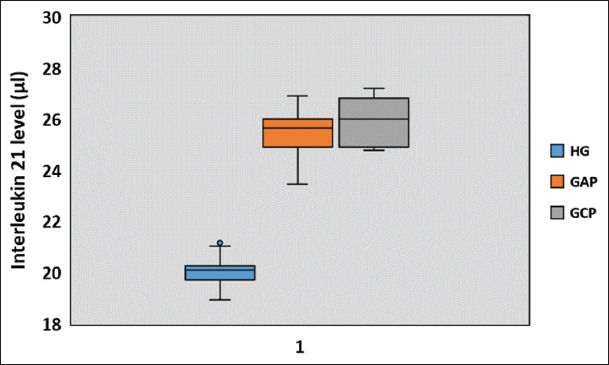
Box plot showing comparison of interleukin-21 (IL-21) levels within groups. IL-21 was highest in GCP, followed by GAP, and then the HG. GCP: Generalized chronic periodontitis, GAP: Generalized aggressive periodontitis, HG: Healthy gingiva.

**Figure 3:**
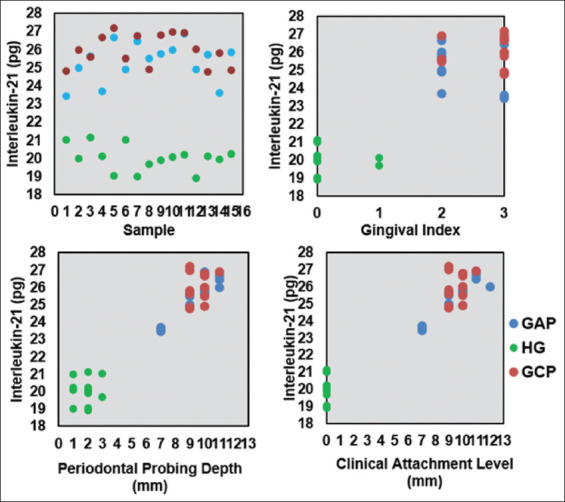
Scatter plot showing interleukin-21 (IL-21) distribution and its correlation with clinical periodontal parameters. Figure shows correlation of individual periodontal parameter with IL-21. IL-21 was consistently increased with increasing pocket depth and attachment loss. HG: Healthy gingiva, GAP: Generalized aggressive periodontitis, GCP: Generalized chronic periodontitis.

Box plot (Figure 2) shows levels of IL-21 in all the three groups. The results show similar IL-21 levels between GAP (25.3±1.1) and GCP (25.9±0.9) groups. Overall comparisons within and between groups for IL-21 using ANOVA showed a statistically significant interaction between the three groups (*P*<0.001). A *post hoc* Bonferroni assessment showed that IL-21 was significantly higher in GCF of GCP (*P*<0.005) and GAP (*P*<0.005) compared to HG controls (20.0±0.7), however, no significant difference was observed between the GCP and GAP groups (*P*>0.05) (Table 2). IL-21 was highest in GCP and higher in GAP compared to HG group. Pearson’s correlation analysis was performed to check the correlation of IL-21 with periodontal parameters (Table 3). A strong positive correlation of IL-21 was found with periodontal PD (PPD) (*r*= 0.966) and CAL (*r*=0.933) in GAP group; and with PPD (*r*=0.924) and CAL (*r*=0.963) in GCP group (Table 2 and Figure 3). A difference between clinical periodontal parameters was calculated using Bonferroni *post hoc* comparison. PI was significantly different between GAP versus GCP (*P*<0.005) and GCP versus HG (*P*<0.005). GI was significantly different between GAP versus GCP (*P*<0.005), GAP versus HG (*P*<0.005), and GCP versus HG (*P*<0.005). The difference in PPD was statistically significant between GAP versus HG (*P*<0.005) and GCP versus HG (*P*<0.005). CAL was statistically significant between GAP versus HG (*P*<0.005) and GCP versus HG (*P*<0.005).

**Table 2:** Bonferroni *post hoc* pairwise comparison of interleukin-21 levels between groups

A	B	Mean difference (A-B)	Std. error	*P*-value	95% confidence interval	

					Lower bound	Upper bound
HG	GAP	−5.31	0.33	**0.000**	−6.12	−4.49
	GCP	−5.94	0.33	**0.000**	−6.76	−5.13
GAP	HG	5.31	0.33	**0.000**	4.49	6.12
	GCP	−0.64	0.33	**0.176**	−1.45	0.18
GCP	HG	5.94	0.33	**0.000**	5.13	6.76
	GAP	0.64	0.33	**0.176**	−0.18	1.45

Statistically significant *P* values are marked as bold text. HG: Healthy gingiva, GAP: Generalized aggressive periodontitis, GCP: Generalized chronic periodontitis.

**Table 3:** Pairwise comparison and clinical parameter correlations of IL-21 levels

Clinical parameters	Outcome parameter	IL-21 level		

		HG	GAP	GCP
PI	Correlation	0.07	0.08	0.03
	*P*-value	**0.80**	**0.75**	**0.91**
GI	Correlation	0.03	0.13	0.45
	*P*-value	**0.91**	**0.13**	**0.09**
PD (mm)	Correlation	0.25	0.96	0.92
	*P*-value	**0.37**	**<0.05**	**<0.05**
CAL (mm)	Correlation	0.49	0.93	0.96
	*P*-value	**0.07**	**<0.05**	**<0.05**

Statistically significant *P-*values are marked as bold text. IL-21: Interleukin-21, HG: Healthy gingiva, GAP: Generalized aggressive periodontitis, GCP: Generalized chronic periodontitis, PI: Plaque index, GI: Gingival index, PD: Probing depth, CAL: Clinical attachment level.

## 4. Discussion

The aim of this investigation was to compare IL-21 levels in GCF samples from patients with GCP, GAP, and HG and to correlate IL-21 levels with conventional clinical parameters. The results show that IL-21 levels are elevated in both GCP and GAP and that a positive correlation exists with PD and CAL parameters. In general, once the active innate host defense mechanism is breached bacteria enter the underlying tissues. On encounter with antigens by cells in deeper tissues the adaptive immune system is activated. Mediators of adaptive immunity such as B cells, T cells, and Th cells are critical for immune reaction and immunoregulation [39]. Among the Th subsets, Th17 is a relatively new subset executing inflammation in infective, autoimmune, and osteolytic pathogenic pathways in several systemic diseases [40-43]. Our interest in the cytopathogenesis of IL-21 in inflammation was strengthened by its association with various inflammatory conditions. Hence, this cross-sectional study was undertaken to compare and quantify IL-21 among healthy and periodontally affected gingiva.

Previously reported overexpression of Th17 cytokines (IL-17, IL-21, and IL-23) in chronic periodontitis patients in Brazilian and Chilean population have been well documented and indicates that Th17 facilitates the progression of inflammation [33,44-46]. Several studies in addition to our data reveal a positive correlation of IL-21 to clinical periodontal parameters, thereby strengthening evidence of its role in periodontitis pathophysiology. Similarly, in concordance with previous literature, our data indicate significantly elevated IL-21 in GCP compared to HG, however, the quantity of detected IL-21 in our study (South Indian population) was lower compared to other studies [33,34,44]. Possible explanation may be due to largely diluted crevicular biomarkers as a result of increased GCF volume in severely inflamed crevices of GAP and GCP compared to HG, causing low detection due to high consumption of IL-21 in inflammatory pathogenesis [47]. Since most of cases included in this study had severe inflammation and bone loss (i.e., PD), this could justify the low detection of IL-21. Another consideration is the possibility of racial differences in inflammatory cytokine level modulation [48].

Supplemental to the pathogenic rationale of IL-21 in GCP mentioned in the past literature, other IL-21 mediated mechanisms could be stipulated in periodontal destruction and its occurrence around clinically healthy teeth. Initiation of periodontal and periapical bone destruction is mediated by stimulation of osteoclasts and key bone resorptive factor receptors like receptor activator of nuclear factor k-B ligand, which are upregulated in the presence of Th17-related cytokines [49-51]. IL-21 also stimulates fibroblastic MMPs and prostaglandins to initiate soft-tissue dissolution and ensures the disease destructive process [45].

**Figure 4:**
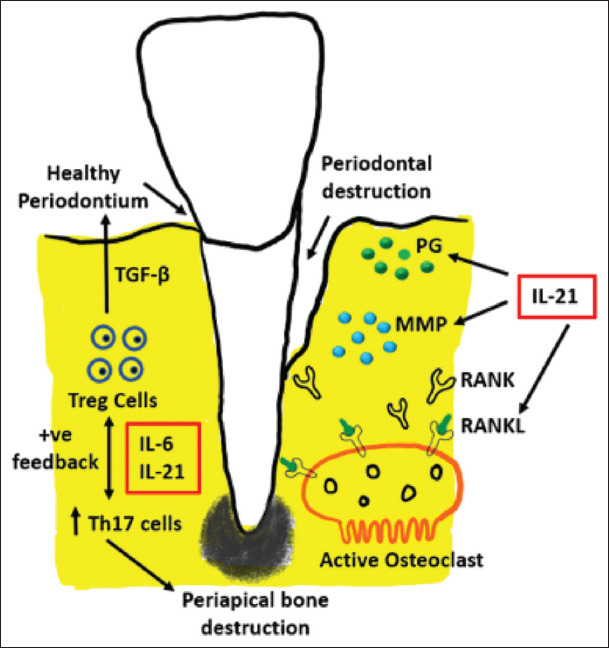
Mechanism of IL-21-mediated bone destruction and soft-tissue dissolution. IL: interleukin, TGF-β: Transforming growth factor-beta, PG: Prostaglandin, MMP: Matrix metalloproteinase, RANK: Receptor activator of nuclear factor κ-B, RANKL: Receptor activator of nuclear factor κ-B ligand.

Next to local tissue breakdown, local chemical imbalance also perpetuates disease progression. Treg cells are responsible for periodontal homeostasis and are normally influenced by transforming growth factor-beta (TGF-β) (anti-inflammatory and immunosuppressive cytokine). However, with elevated IL-6 or IL-21 profiles, TGF-β increases Th17 differentiation leading to Treg underdevelopment and Th17 overexpansion [46,52] (Figure 4). Our study provided new data showing a significant increase of GCF IL-21 in GAP compared to HG. The propensity of IL-21 to modulate PMNs in inflammation has been reported [53]. Overexpression of Th17 impairs the adhesion between ICAM-1 and LAF-1, inhibiting neutrophil extravasation [54]. In the locally deranged host barrier region, IL-21 in GAP may further alter the efficiency of PMNs in microbial defense against subgingival microflora. Alternatively, overaccumulation of PMNs locally may cause uncontrollable tissue destruction. Ineffective clearance of harmful periodontopathic bacteria leads to microbial persistence and inflammatory progression. Although elevated IL-21 levels in GCF of GAP were reported for the 1^st^ time, no significant difference in IL-21 levels was found between GCP and GAP. Interestingly, similar results were reported previously in saliva and serum samples [31]. Several theories have been proposed in the past claiming no difference between cytokine milieu and chemical profile between GCP and GAP subjects. One theory proposes that local tissue insult occurs over a prolonged period of time in GCP, while there is an acute host response manifesting clinically as severe tissue destruction in GAP. Pathological destruction exists in a continuous phase followed by a phase without destruction, this was explained by the “continuous and episodic burst model of periodontitis” [55]. Long-standing GAP cases gradually turn into chronic inflammatory periodontal conditions, which may explain no differences between IL-21 levels in the GAP and GCP groups [56]. Similar scenarios were reported by Mattuella *et al*. with no difference in Th1/2-related cytokine profiles in GCP and GAP groups. Similarly, in a detailed review by Duarte *et al.*, no difference was reported in cytokine profile of GCP and GAP groups [30,57]. The latter reported that IL-1β and IL-6 levels were similar in the GAP and GCP groups, and it was already known that IL-1β, IL-6, and IL-21 aid T cells and B cells in the development of antibody response through similar transcriptional mechanisms. IL-1β and IL-6 have upregulating effects on IL-21 and it makes sense that a certain amount of IL-6 can only generate a proportionate supply of IL-21. Current literature suggests that no difference in infection ratios may exist between GAP and GCP and that they yield similar chemokine profiles in GCF occasionally [30]. These studies report similar IL-21 levels in both GAP and GCP groups as reported in our study.

IL-21 was also found in GCF of healthy subjects in our study. This was reported in healthy subjects by other groups previously [33-36]. A recent study reported in an experimental animal model that IL-21 along with anti-Tim1 (T cell immunoglobulin and mucin domain) and CD40L treatment enhanced B10 cells capability of producing anti-inflammatory cytokine IL-10, which inhibited periodontal bone loss [58]. Another study reported that IL-21 induced IL-10 mRNA and protein synthesis, which could suggest its indirect role in immunosuppressive activity [59]. Findings from these studies support the results of our study by further arguing the possibility of IL-21 as having potential anti-inflammatory activity.

There are some points that should be considered regarding the present investigation. A small study population and a lack of microbiological diagnosis of periodontitis are two important limitations of this study. Increased IL-21 in periodontitis explains its synergistic destructive capability with cocontributing factors, whereas its presence in healthy tissue reveals its protective role. However, delineation up to which level IL-21 has an anti-inflammatory effect and beyond which it has a pro-inflammatory role are still debatable. Meanwhile, it would be interesting to see if there are equal degrees of shift in concentrations of IL-21 levels in GCP and GAP groups following periodontal therapy. Data from such studies may support and strengthen evidence to classify IL-21 as a biomarker in periodontitis and would clarify its role in the balance between Tregs, Th17 cells, Th2 cells, and their crosstalk in the pathophysiology associated with tissue inflammatory destruction.

## 5. Conclusion

Although periodontitis pathophysiology involves complex interplay between pro-and anti-inflammatory signaling, data on IL-21 revealed elevated levels in both GCP and GAP. Further longitudinal studies are required to further characterize and determine the diagnostic value of IL-21 as a reliable biomarker in periodontal disease.
